# Molecularly Imprinted Polymer (MIP)-Based Electrochemical Sensors for Pharmaceutical Detection in Wastewater: Imprinting Strategies, Analytical Performance, and Challenges

**DOI:** 10.3390/s26113600

**Published:** 2026-06-05

**Authors:** Hopewell Mnyandu, Precious Mahlambi, Mun’delanji C. Vestergaard

**Affiliations:** 1 Department of Chemistry, University of KwaZulu-Natal, Private Bag X01, Scottsville, Pietermaritzburg 3209, South Africa; 212539071@stu.ukzn.ac.za (H.M.); mahlambip@ukzn.ac.za (P.M.); 2 Department of Food Science and Technology, Faculty of Agriculture, United Graduate School of Agricultural Sciences, Kagoshima University, 1-21-24 Korimoto, Kagoshima City 890-0065, Japan

**Keywords:** molecularly imprinted polymers, electrochemical sensing, pharmaceutical contaminants, wastewater monitoring, voltammetry, impedance spectroscopy, environmental analysis

## Abstract

Molecularly imprinted polymers (MIPs) have emerged as robust and versatile recognition elements for electrochemical sensing due to their high chemical and mechanical stability, cost-effective fabrication, and excellent selectivity toward target analytes. In recent years, MIP-based electrochemical sensors have gained significant attention for the detection of pharmaceutical contaminants in wastewater, addressing growing environmental and public health concerns. This review provides a comprehensive overview of the fundamental principles of molecular imprinting Emphasis is placed on fabrication strategies and electrochemical detection techniques, including cyclic voltammetry, differential pulse voltammetry, and electrochemical impedance spectroscopy. Furthermore, it discusses imprinting mechanisms for different classes of contaminants, matrix effects, and other challenges. By critically analyzing recent applications, this work highlights key factors influencing sensor performance, such as sensitivity, selectivity, and detection limits. Finally, we touch on future perspectives, focusing on the development of more reliable, scalable, and environmentally sustainable sensing platforms for real-world wastewater monitoring.

## 1. Introduction

Molecularly imprinted polymers (MIPs) are receptor-like materials that are chemically synthesized to specifically target and bind analytes (also called templates) of interest [1]. MIPs are designed via a lock-and-key principle that is usually observed in the enzyme–substrate complex; hence, MIPs are considered synthetic or artificial receptors since they can mimic functions of a natural receptor such as enzymes, antibodies, antigens, etc. [2]. They possess properties such as inexpensiveness, ease of fabrication, robustness, specificity, and high affinity; hence, they have gained more interest than natural receptors [3]. Moreover, MIPs have long shelf lives even at ambient conditions, and they can withstand extreme environments, basic or acidic conditions, high temperatures, and high pressures [4].

**Figure 1:**
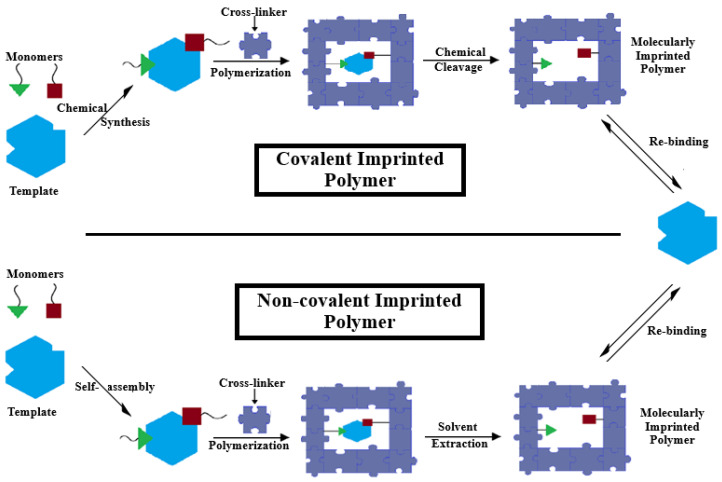
Covalent and non-covalent mechanisms for the formation of a molecularly imprinted polymer (MIP).

The MIPs are fabricated by using either covalent or non-covalent (hydrogen bond, ionic, or hydrophobic) interactions during pre-polymerization of the template molecule/target analyte with functional monomers. Then, a cross-linking molecule is added with an initiator under photo/thermal conditions, creating a polymer knitting that is formed around the complex [5]. The template molecule is thereafter removed via solvent elution since the template molecules are very soluble in solvents [5]. This leaves the complex with cavities that are comparable with the template molecule in terms of shape, size, and chemical functionality distribution [6]. This suggests that the MIP complexes can selectively rebind the target analyte when immersed in a solution containing the target analyte (such as pharmaceutical drugs) and the interfering substances [5]. Figure 1 shows a schematic diagram illustrating the formation of a molecularly imprinted polymer (MIP).

Pharmaceuticals are natural or developed compounds that can cure, diagnose, prevent, or even treat diseases in humans and animals. They are designed to be biologically active and persistent at low concentrations to have pharmacological impact and therapeutic activity [7,8]. Pharmaceutical compounds include, among others, antibiotics, antiretroviral drugs (ARVDs), nonsteroidal anti-inflammatory drugs (NSAIDs), and antiepileptic drugs (AEDs) [9]. Pharmaceutical compounds have been considered emerging environmental (water) contaminants in the last few decades due to their potential harmful health impacts on biotic communities [9,10]. Pharmaceutical contaminants in water bodies continue to accumulate at an increasing rate, and the leading contributors are wastewater treatment plants (WWTPs) [7,11]. This is due to the WWTP processes failing to eliminate pharmaceutical substances completely. Thus, they are released with WWTP effluents into the nearby surface waters [7,11].

Pharmaceutical drugs’ accumulation in the environment increases with increasing drug production and consumption, and poor waste management [12]. Drugs are produced to have pharmacological impact at very low dosages, and to be stable enough to reach and interact with the intended target molecules [12]. However, they are also able to produce ecotoxicological impacts on plants, microorganisms, animals, and human health due to accumulation [13]. It has been reported that drugs consumed by humans can be excreted with urine and feces. They are excreted unaltered or in a partially metabolized form, in the range 30–90% [13].

As examples of the degradation of parent drugs, carbamazepine undergoes natural attenuation or hydrolysis to form carbamazepine-10,11-epoxide and other intermediates; ibuprofen undergoes biodegradation to form hydroxy-ibuprofen metabolites [14,15,16]; and diclofenac undergoes photolysis or biodegradation to form 4′-hydroxy diclofenac and other chlorine-lost products [16,17]. However, pharmaceutical drugs can persist and remain unchanged for more than 10 years in the environment [13]. It is because of these pressing problems caused by increase in use of pharmaceutical drugs coupled with poor management once they are discharged into the waste waters that technologies for efficient and effective management of this growing threat are required.

Molecularly imprinted polymers (MIPs) is a technology that is suitable for managing contamination of pharmaceuticals in wastewater. MIPs have various applications, including as a sample preparation tool in bioanalytical techniques, in electrochemical sensing, in catalysis, drug delivery, and chromatography, and as sorbent beds in solid-phase extraction [18,19]. Thus, MIPs offer flexibility and are robust. One of the most crucial aspects is that they can easily be coupled with instruments such as electrochemical devices. Although different types of analytical techniques such as spectroscopic, spectrophotometric, and chromatographic have been extensively employed for the detection of pharmaceutical contaminants [20,21], and are powerful, accurate, and precise, they have some drawbacks. They are tedious and expensive, and employ relatively expensive instruments with complicated operating steps that require qualified personnel [21]. As such, these analytical techniques cannot be utilized on a large scale in the environment to monitor contaminants for preventative or regulatory actions [20]. Therefore, there is a need for a technique that is simple, cheap, and user-friendly but has high sensitivity and a fast response time, and can be fabricated into a smaller portable analytical device for use in the field. MIPs coupled with electrochemical devices offer a viable solution.

Electrochemical techniques offer all the advantages that other analytical tools like spectroscopy do, but are relatively cheaper, and can be miniaturized and used in the field. One of the electrochemical techniques that has been widely used is voltammetry. However, voltammetric analyses have low selectivity to target molecules when used without recognition molecules, making it difficult to ensure selectivity when applied to complex matrices with interfering substances [21]. Therefore, incorporating a selective adsorbate onto the surface of the working electrode improves the electrochemical technique’s selectivity towards the target analyte [21]. The adsorbate must be a specific receptor layer with high affinity for target molecules and limited cross-selectivity [20]. Therefore, MIP-integrated electrochemical sensing has gained attention over the years. Furthermore, they offer ease in miniaturization; have rapid response, cost-effectiveness, and portability; and have very good detection capability in terms of sensitivity [22].

Despite the aforementioned advantages, MIPs lack conductivity and electrocatalytic activity. Consequently, researchers have utilized and continue to utilize different types of nanomaterials to enhance MIP conductivity and electrocatalytic activity [23]. The nanomaterials include quantum dots (QDs), carbon-based materials, metal oxides, metal–organic frameworks (MOFs), magnetic nanoparticles, nanowires, nanorods, nanofibers, polymeric nanomaterials, etc. [24]. For example: Zhong et al. [25], developed a molecularly imprinted electrochemical sensor by incorporating gold nanoparticles onto a glassy carbon electrode (GCE) via electrodeposition for the detection of neutral phosmet residues. The sensor showed improved electrocatalytic activity and conductivity over MIPs alone; within a linear range of 0.020 to 4.0 nmol/L, the sensor gave a very low detection limit of 0.010 nmol/L compared to 0.17 nmol/L obtained from the MIP coupled with GCE [25]. Dong et al. [26] produced a graphene/reduced graphene oxide (rGO)-based MIP sensor for the detection of testosterone, diclofenac, and other targets, where graphene oxide and rGO were incorporated onto the surface of GCE prior to MIP deposition. An improvement in terms of current response and detection limits was observed.

It can be noted that MIPs combined with electrochemical techniques and different types of nanomaterials can outperform other analytical methods in terms of selectivity, sensitivity, and robustness [24].

A typical MIP-based electrochemical sensor consists of polymer recognition as a bioreceptor or mediator of electron transfer during the redox reaction of the target analyte [27,28]. Biochemical recognition molecules that improve the electrochemical sensor’s selectivity towards the target analyte tend to lack electrochemical response. That is, they have poor electrocatalytic activity. Therefore, there is a need to incorporate recognition molecules with materials (for example, metals, metallic compounds, carbon nanomaterials, nanocomposites, etc.) that have electrocatalytic properties, strong adsorption capacity, and relatively large surface area [28,29,30]. On having relatively large surface area, nanomaterials are most attractive because they can improve detection sensitivity even more [28,31].

This review focuses on recent developments; sensing efficiency; ongoing issues like selectivity and matrix interference; and future outlooks for enhancing sensitive, swift, and affordable environmental monitoring of the synthesis of MIP-based electrochemical sensors and their applications.

## 2. Different Types of Imprinting Mechanisms for Different Classes of Contaminants

### 2.1. Non-Covalent Imprinting

Non-covalent imprinting requires less synthesis effort and has easy template removal or binding due to weak bonds between the polymer network and the template [32]. This method works well with environmental samples and depends on hydrogen bonding, π–π interactions, and van der Waals forces. Polar and slightly polar pharmaceutical drugs, such as antibiotics, non-steroidal anti-inflammatory drugs (NSAIDs), and beta-blockers, which are denoted by H-bond donors or acceptors, are best suited for non-covalent imprinting. This method is extremely compatible with aqueous and mixed solvents; it is known to produce heterogeneous cavities (recognition sites). However, its efficacy is compromised when utilized in aqueous matrices because there may be competition caused by water, which competes for hydrogen bonding and inhibits the template–monomer interactions [33,34,35]. Non-covalent imprinting can produce both template-specific recognition sites and non-specific binding sites but more of the non-specific binding sites (a limitation) due to the use of large amounts of functional monomers during polymerization [32].

### 2.2. Ionic (Electrostatic) Imprinting

This method depends on the strong electrostatic interaction between the template and the monomer, resulting in enhanced selectivity in aqueous media. This method is best suited for polar chiral templates (beta-blockers such as the eutomer of atenolol), antidepressants, and NSAIDs, especially acidic/basic pharmaceuticals (charged-ionizable drugs). However, this method’s effectiveness is limited by the interference from the ions present in complex matrices such as wastewater, which causes competition, and the method depends on high pH and ionic strength [36,37].

### 2.3. Metal Ion-Mediated Imprinting

The use of Fe^3+^/Cu^2+^ bridging-mediated MIPs promotes strong and directional coordination bonds with chelating pharmaceutical drugs such as tetracyclines and fluoroquinolones. This method is best suited for environmental remediation, as it provides highly stable and selective MIPs for metal-binding pharmaceutical contaminants. However, there are challenges associated with the metal ion-mediated imprinting method. These include the loss of metal ions through leaching, competition from interfering ligands, and changes in the sensor’s response due to other components present in complex matrices (e.g., wastewater samples) instead of the analyte itself [30,37,38].

### 2.4. Hydrophobic Imprinting

Non-polar drugs and aromatic compounds such as endocrine disruptors (e.g., estradiol and bisphenol A), steroids/hormones, and hydrophobic drugs are the best-suited template molecules in hydrophobic imprinting [38,39,40]]. This method can be effectively utilized for matrices that are aqueous since there is less interference from water. However, this method can be problematic due to its producing MIPs that lack specification and have weak chemical bonds compared to ionic or hydrogen interactions, making it very difficult to monitor contaminants present in water matrices [38,39,40].

### 2.5. Covalent Imprinting

The method has high precision since it can produce MIPs with homogenous binding sites of high specificity due to targeting the small, rigid analytes (e.g., boronic ester or small-Schiff-base pharmaceutical analogs). Therefore, the method has minimal non-specific binding. A large amount of cross-linking agent is required to achieve well-defined binding cavities with complementary steric and functional topography to the template molecule [32]. This can improve the produced MIP in terms of selectivity, structure rigidness, shape memory, and chemical and thermal stability (solvent, pH changes, temperature), etc. [41,42,43]. This is best suited for catalysis [32]. One of the method’s limitations is having to bind very small amounts of target analyte due to fewer available cavities, resulting in slower binding kinetics. This issue is associated with stubborn template removal; thus, it is poor in applicability [44,45]. Another disadvantage of covalent imprinting is that it is only restricted to alcohol, amine, aldehyde, ketone, or carboxylic acid functional monomers/templates [32].

### 2.6. Surface Imprinting (with Non-Covalent/Ionic)

This method is best suited for large/complex macromolecules such as proteins, peptides, and biomacromolecules (e.g., bovine serum albumin, biomarkers, etc.), since most of the binding sites are easily accessible. This then promotes faster binding kinetics and enhances sensor performance. The drawback of this method is the lower binding capacity surface instability; also, it is complicated to synthesize the MIP [46].

In summary, there is a need for improving binding stability and selectivity within complex matrices such as wastewater, especially on techniques for non-covalent and ionic imprinting. This is because the mentioned techniques are strongly affected by pH changes, interfering ions, and hydrogen bonding competition from interfering species. Hence, more research needs to be directed towards the development of stable MIPs that exhibit homogeneous binding sites and less impact from fouling and leaching, and MIPs that are stable and robust, have faster-binding kinetics, and have universal applications, particularly for larger biomolecules and different classes of pharmaceutical pollutants present in the environment.

## 3. Different Types of Electrochemical Techniques for MIP-Based Sensing in Complex Water Matrices

### 3.1. Differential Pulse Voltammetry (DPV)

Differential pulse voltammetry is one of the sensitive electrochemical techniques used in biosensing and chemical sensing [47]. The improved sensitivity is associated with interfacial capacitance, which suggests that the current response is less susceptible to interference arising from small variations in the applied potential [47]. DPV is recommended for the detection (trace analysis) of heavy metals, antibiotics, and pesticides in complex sample matrices like wastewater [48,49]. DPV offers excellent signal-to-noise ratios and high sensitivity; however, calibration in real sample matrices remains a challenge. The DPV response can be compromised due to the interferences of co-existing species in the sample or due to electrode fouling [48,49].

### 3.2. Square Wave Voltammetry (SWV)

SWV is a sensitive electroanalytical technique used to measure redox (electron-transfer) processes of electroactive analytes by differentiating Faradic processes from charging currents [50]. This technique is fast in response (compared to DPV), highly sensitive, and can easily separate faradaic currents (redox) from unreliable background currents. Furthermore, this technique is ideal for multi-analyte detection of pharmaceuticals and pollutants in complex mixtures/matrices such as wastewater and surface water samples [48]. The SWV technique can be impacted by matrix effect and electrode fouling, and requires optimization of the frequency/amplitude [48].

### 3.3. Stripping Voltammetry

This is an electroanalytical technique that initially promotes the target analyte being electrodeposited onto the surface of the electrode and applies a potential sweep to promote a redox reaction of the analyte and measure the current response [51]. The measured current is proportional to the concentration of the analyte present. Stripping voltammetry is highly effective for ultra-trace metal analysis in environmental waters because preconcentration of analytes yields exceptionally low limits of detection (LODs) [49]. Stripping voltammetry can be categorized into three techniques, which are anodic stripping voltammetry (ASV), cathodic stripping voltammetry (CSV), and adsorptive stripping voltammetry (AdSV). ASV is the common electroanalytical approach because it is highly sensitive [51]. This method begins with the reduction and deposition of the analyte onto the electrode surface. A positive-going (anodic) potential sweep is then applied to oxidize the analyte, creating an anodic signal from which the analyte concentration is derived [51]. CSV does the opposite: The analyte is first oxidized and deposited onto the electrode, then a positive (cathodic) potential sweep is applied to reduce the analyte. Subsequently, the response is measured as the current or concentration of the analyte being stripped. AdSV allows for the adsorption of the electroactive analyte rather than the electrodeposition. The accumulated analyte on the surface is then subjected to a cathodic scan (reduction or decomposition), resulting in a current response that is proportional to the total concentration of the analyte [51]. Stripping voltammetry is heavily limited by organic matter interference, as the electrode’s surface is prone to poisoning; thus it requires precise deposition conditions [49].

### 3.4. Electrochemical Impedance Spectroscopy (EIS)

EIS is an electroanalytical technique which can measure how a system resists and responds to the application of a small AC signal over a range of frequencies. EIS analysis shows the interfacial properties (migration, charge-transfer/mass-transfer, diffusion, and intercalation processes) from the bulk solution to the electrode surface. This technique can be used for pharmaceuticals, biomolecules, and pathogens, and it is highly effective even when applied to turbid/colored samples such as wastewater [52]. It is very useful for sensing technologies including MIPs and biosensors. EIS can be used to analyze material properties and processes that can affect the conductance, resistance, or capacitance of the electrochemical cell [52]. Despite its advantages, EIS is limited by non-specific adsorption effects that can reduce selectivity, lengthy measurement times, complex data analysis, and the need for a stable experimental setup. Despite its advantages, EIS is limited by non-specific adsorption effects that can reduce selectivity; lengthy measurement times; complex data analysis; and the need for a stable experimental setup [53,54].

### 3.5. Cyclic Voltammetry (CV)

CV is a technique used to study electron-transfer or redox behavior of redox-active substances including pharmaceuticals, drugs, and other pollutants [49,55]. In CV, the potential at the working electrode is swept linearly between two predetermined values and then reversed, while the resulting current from the redox reaction is recorded. CV is best suited for sensor characterization; however, it is not recommended for trace quantification due to poor selectivity in complex mixtures and low sensitivity. Another drawback of CV is the generation of high capacitive current, which is not ideal, since it tends to interfere with the faradaic currents that are the response from the actual redox reaction of the target molecule species [49,55].

### 3.6. Potentiometry

Potentiometry uses a high-impedance voltmeter to measure the electrochemical potential difference between two electrodes with no significant charge carrier (electrical current) flowing across them [56]. This electroanalytical technique is ideal for the determination of ions, including nitrate, ammonium, and pharmaceuticals in ionic form, in ion-selective electrode (ISE)-based sensors [57]. The technique is simple, budget-friendly, and suited for the analysis of wastewater/turbid water (complex matrices) due to it being selective for specific ions. However, the technique has poor sensitivity towards organics and is limited to ionic species, and its electrode’s potential gradually changes (drift over time) even if the analyte concentration remains the same [57].

### 3.7. Amperometry/Chronoamperometry

Amperometry measures the current (*i*) over time (*t*) resulting from the oxidation or reduction of electroactive biomolecules at a constant applied potential [58]. Chronoamperometry specifically measures this temporal current response after applying a potential step to the working electrode. In both cases, the redox rate and current magnitude are directly determined by the analyte concentration [59]. The techniques are fast and suitable for continuous monitoring and do not need complicated instrumentation or operation. They are recommended for biosensors and flow systems since they can do real-time analysis for pharmaceuticals, glucose, phenols, etc. The drawbacks of these techniques include poor selectivity, susceptibility to surface fouling and drift (change in the measured current over time), and high impact by interfering electroactive species [48].

The most effective electroanalytical techniques are differential pulse voltammetry (DPV), square wave voltammetry (SWV), and stripping voltammetry (ASV/CSV/AdSV). This is due to that they can produce very low detection limits, indicating that they are highly sensitive; thus, they can detect contaminants (pharmaceuticals and heavy metals) at trace levels. Electroanalytical techniques such as electrochemical impedance spectroscopy (EIS), amperometry, and potentiometry have great potential in real-time environmental monitoring and direct detection of target analytes without the addition of markers. Cyclic voltammetry still stands out as an ideal technique for sensor (electrode) characterization. More investigation is required for improving issues of surface (electrode) fouling and matrix effects from interferences and to ensure calibration with complex matrices (e.g., wastewater). These improvements should focus on integrating MIPs with nanomaterials and real-time applications of sensing platforms (portable devices) for onsite environmental monitoring. The table below presents the overview of molecularly imprinted polymer (MIP)-based technologies: sensing mechanisms, target analytes, advantages, and limitations: MIP in sensing technology has advanced dramatically, especially in separation, catalysis, and drug delivery. MIPs possess high selectivity, stability, and variable recognition properties. In sensing techniques such as potentiometric, voltammetry, DPV, and impedimetry, their application extends to pharmaceuticals and environmental pollutants or trace pollutants. Even though these techniques have drawbacks such as fouling, matrix effects, and limited use to certain receptors, they are still advancing in electrochemical sensing technology. Optical MIP sensors can be used beyond detection analysis; however, the technique is still not cost-effective, as it requires labels/markers. Beyond sensing, MIPs show strong potential in controlled drug delivery, enzyme-like catalysis, and selective separation processes. They offer performance in complex environments; however, method optimization is still required to overcome these limitations of biocompatibility, catalytic efficiency, and template leakage. To show the diverse application of MIP in sensing technology, Table 1 represents a comparative overview of major MIP-based technologies, target analytes, chemical mechanisms, advantages, and their limitations.

## 4. Impact of Matrix Effects on the Performance and Practical Application of MIP-Based Electrochemical Sensors

Matrix effects are known to impact significantly the practical application of MIP-based sensors in real samples of complex matrices (e.g., wastewater, food, environmental or biological samples). Different components that are commonly found in these types of samples include proteins, salts, surfactants, organic matter, metal ions, suspended particles, dissolved organic matter, etc. They tend to alter the electrochemical mechanism occurring at the transducer interface, such as electron-transfer kinetics, diffusion of the redox probe at the electrode surface, and the analyte binding mechanism. However, these interfering components can affect the performance of the sensor through signal instability, electrode fouling, reduced binding efficiency, lower selectivity, and inaccurate analytical responses [69,70,71]. Table 2 shows different types of matrix effects from commonly present components in environmental samples which impact the performance of the sensor.

Matrix effects continue to have a significant impact on the practical use of MIP-based electrochemical sensors because they can impair sensor response in terms of signal stability, electron-transfer mechanisms, and binding efficiency. For reliable real-sample analysis, these interferences must be addressed by improved sensor engineering (electrode modification), taking into account matrix effect in the sensor design.

## 5. Current Limitations and Emerging Challenges in Electrochemical Sensing

It is essential for developers to understand challenges or limitations associated with electrochemical sensor technologies in order to design and fabricate durable and reliable electrochemical sensing technologies. These sensing technologies must be able to withstand complications that result from environmental, biological, biomedical, food safety, and industrial analysis applications. These challenges include electrode fouling, matrix effect, signal drift, poor selectivity, and mechanical and structural instability.

Electrode fouling is caused by the gradual buildup of components on the surface of the transducer, such as products from chemical reactions, matrix substances, organic matter, microorganisms, proteins, etc., from wastewater, biological fluids, and environmental samples [80,81,82]. This buildup is associated with inhibition of electron transfer when the electrode surface is blocked. The potential impact could be a signal shift, reduced sensitivity, shorter shelf life of the sensor, and poor reproducibility [80,81,82]. The mass transport limitations are often caused by slower analyte diffusion towards the electrode surface. This can limit reaction rates due to inadequate mass transfer, resulting in poor sensitivity and slow transducer responses [83,84]. This is more prominent when the analyte concentration is very low in the system. Some analytes that lack energy or electrochemical activity, or have slow electrochemical reactions may produce low current responses (decreased sensitivity); therefore, the transducer may require additional surface modification [83,84].

The **matrix effect** from the presence of salts, proteins, surfactants, metal ions, dissolved organic matter, and suspended particles could impact sensor performance. Since electron transfer kinetics can vary, causing a signal decrease or increase, this impacts the accuracy as the response generated is a faulty one [81,83,84]. Many interfering substances from samples such as food extracts, river water, blood, and urine could lead to poor sensor selectivity towards the target, especially if the substances are electroactive. This can lead to overlapping signals (voltammetric) and false positives, and reduced analytical reliability [83,84,85].

**Signal drift** is a common issue that is associated with transducer aging; material degradation; accumulation of molecules, which causes electrode fouling; and variation in the environmental conditions. This can result in poor reproducibility, and inaccuracy. As a result, there is need for constant recalibration, increasing maintenance requirements [85,86].

The **stability** of electrochemical sensors can degrade over time if the sensing technology includes modifiers such as nanomaterials, aptamers, polymers, enzymes, and other surface modifiers. When these materials interact with real samples, they may lead to chemical degradation, drifting of sensing layers, chemical reactions, and loss of activity, resulting in poor long-term sensor stability [86,87]. This impacts commercial viability and utilization in the field [83,87]. It is hard to fabricate a sensor with low **detection limits** while having a stable and selective sensor. Therefore, the analysis of various pollutants, pharmaceuticals, hormones, and biomarkers is very complex because they exist in the environment at very low concentrations (trace ng/L-µg/L levels) [83,88].

Even though electrochemical sensors show great analytical performance at a laboratory scale, they can pose a challenge in **commercialization or large-scale upscaling**. The transition from laboratory sensors to market-ready sensors is limited by manufacturing variability, quality control issues, sensor shelf-life, and stability and regulatory requirements [85,86]. **Cost and instrumentation complexity** in the advancement of electrochemical sensors also pose a challenge due to factors such as the cost of nanomaterials, complicated fabrication processes and advanced instrumentation, the cost of mass production, and limited large-scale adoption [84,89]. Furthermore, **lack of standardization** is another major concern leading up to the above challenges because of differences in fabrication techniques, testing protocols, electrode materials, and reporting standards according to different studies and laboratories across the globe [86].

The above-mentioned challenges are significant, especially in the environmental analysis of pharmaceuticals, including antibiotics and antiretroviral drugs, in complex matrices such as rivers or wastewater. However, the advancement of electrochemical sensors has gained more attention in recent years, since they are relatively cost-effective, provide fast responses, and have high selectivity and sensitivity [3,26,30]. The incorporation of materials such as molecularly imprinted polymers (MIPs) has been reported to improve the selectivity of the electrochemical sensors. However, MIPs have poor electrical conductivity, which affects the sensor sensitivity [30]. On the other hand, the incorporation of nanomaterials has shown to improve the sensitivity of electrochemical sensors. This is due to the nanomaterials having a high surface area, which enhance the sensor interaction with the target compound, accompanied by the high electrical conductivity of the nanomaterial, which enhances electron transfer kinetics between the sensor and the target compound [3,26,30]. Thus, the combination of nanomaterials with MIPs provides an electrochemical sensing technology with improved properties such as high sensitivity, selectivity, and electrical conductivity.

In order to overcome the outstanding challenges, it is crucial that advanced electrode materials, antifouling techniques, better sensor designs, standardized procedures, and reliable calibration methods are developed. This will further help in the commercialization and scaling up of electrochemical sensors from the laboratory to real-world monitoring applications.

## 6. Applications for Molecularly Imprinted Polymer-Based Electrochemical Sensors

MIP-based electrochemical sensors are a type of sensor that can detect and quantify target analytes in a sample by combining the selective recognition properties of the MIP at an electrode surface [5]. The MIP-based electrochemical sensor monitors changes in electrical signal response (for example, current, voltage, or impedance) as the template/target analyte selectively binds to imprinted cavities that form molecular recognition sites. The changes in the signal can be monitored and quantified using voltammetry (for example, cyclic voltammetry (CV) and differential pulse voltammetry (DPV), or electrochemical impedance spectroscopy (EIS) [5].

A good MIP-based electrochemical sensor must have the following properties: a selectively modified electrochemical surface must be very sensitive and have a huge surface area, and the template used must be monotonic [61]. Furthermore, it must possess high affinity for the target analyte, and the electrochemical surface must be available for direct contact with the electrode reaction. For voltammetric analyses, it is important to have a redox-active molecule analyte. In a case of a non-redox analyte which cannot produce an electrochemical signal, a molecular electroactive probe must be used to monitor any response due to binding events occurring at the electrode surface [61,90]. The next step in sensor development is surface passivation, which is the formation of an inert thin layer (i.e., oxides) on the electrode surface which suppresses any electrochemical reaction [91]. Surface passivation is a common issue facing non-irreversible drug molecule electrochemistry; thus, the surface of the electrode that is subjected to electrochemical experiments must avoid passivation, and the surface should be easily accessible [61]. In voltammetric sensors, MIP incorporation onto the surface of the working electrode can be achieved either via electropolymerization, grafting, spin-coating, or physiosorption of pre-synthesized MIP suspension [61].

In the last part of this section, we will provide some examples of MIP electrochemical sensor fabrication and their application in water and wastewater, using EIS and voltammetry.

Motia et al. [92], describes an electrochemical sensor based on molecularly imprinted technology developed for the detection of triclosan in wastewater and mineral water. The fabrication of the sensor included the polymerization of an acrylamide/bisacrylamide (monomers) solution containing triclosan linked with carboxylic polyvinyl chloride (cross-linker) onto the surface of a screen-printed gold electrode (Au-SPE). The fabricated electrode’s surface was monitored using cyclic voltammetry, differential pulse voltammetry, electrochemical impedance spectroscopy, atomic force microscopy, and Fourier transform infrared spectroscopy (FTIR). Redox probes of potassium ferrocyanide ([Fe(CN)_6_]^4−^) and potassium ferricyanide ([Fe(CN)_6_]^3−^) were used to act as electron transfer mediators. The electrochemical measurements based on the CV technique were conducted under the following conditions: [Fe(CN)_6_]^3−/4−^ solution concentrations were kept at 5 mM, and the potential range was −0.4 to 0.6 V with the scan rate kept at 30 mV/s. The DPV measurements were conducted in a potential range of −0.2 to 0.4 V at a scan rate of 10 mV/s, and the pulse amplitude was kept at 50 mV. The EIS measurements were conducted at alternating currents in a frequency range of 0.1 Hz to 50 kHz. The triclosan molecule (template) was removed by being oxidized from the MIP film attached to the electrode, thus creating an insulating MIP with cavities. This means that the formed MIP on the surface of the electrode cannot conduct electricity; therefore, this promoted the inclusion of a redox probe for charge transfer between the imprint and the solution containing the target analyte. Various concentrations of triclosan were applied to the sensor, ranging from 0.1 pg/mL to 1 ng/mL. As the concentration increased, the current peak on the DPV voltammogram also increased. The EIS analysis showed Nyquist plots with a trend that showed that the sensor resistances (Z) were very low at high concentrations of triclosan. There is an inversely proportional relationship between the analyte concentration and the sensor’s resistance (Z). The DPV analysis showed corresponding results to the ones obtained above: a current peak increase and a decrease in the sensor resistance. The DPV calibration plots were linear, with a coefficient (R^2^) of 0.99 and a limit of detection (LOD) and limit of quantification (LOQ) of 0.23 pg/mL and 0.78 pg/mL, respectively. EIS studies showed R^2^, LOD, and LOQ of 0.99, 4.7 pg/mL, and 14.26 pg/mL, respectively [92].

Feier et al. [93], developed an electrochemical sensor via electropolymerization of the indole-3-acetic acid (I3AA) (a functional monomer) onto the surface of a glassy carbon electrode (GCE) and a boron-doped diamond electrode (BDDE) to detect cefalexin. Electropolymerization was used with cyclic voltammetry with a potential window kept between −1.6 to +1.6 V and −1.0 to +1.2 V for BDDE and GCE, respectively, while the scan rate of both electrodes was kept at 100 mV/s. The formed nanofilms on the electrodes were characterized using atomic force microscopy (AFM) and a redox probe (potassium ferricyanide [Fe(CN)_6_]^3−/4−^), and the electrochemical response of the sensor was assessed using cyclic voltammetry (CV), differential pulse voltammetry (DPV), and electrochemical impedance spectroscopy (EIS). The electrochemical signal of [Fe(CN)_6_]^3−/4−^ obtained before and after target analyte rebinding was utilized to quantify cefalexin. A logarithmic growth was observed from data obtained from the signal response in relation to the concentration. The data was best plotted in a Freundlich adsorption isotherm, resulting in a linear response for both electrodes, with correlation coefficients of 0.995 and 0.998 for GCE and BDDE, respectively. The limits of detections obtained were 3.2 nM and 4.9 nM for GCE and BDDE, respectively. The relative standard deviations were 4.92% and 2.66% for GCE and BDDE, respectively. The sensor was applied to real samples (river water) where 100 nM of cefalexin was spiked, and recoveries of 106.57% and 107.14 were obtained for GCE and BDDE, respectively [93].

Charkravarthula and Mugweru [94] developed a molecularly imprinted electrochemical sensor where poly-pyrrole was electropolymerized onto a glassy carbon electrode to detect morphine in wastewater samples. The modified electrode was characterized by using cyclic voltammetry (CV) and electrochemical impedance spectroscopy (EIS) with the [Fe(CN)_6_]^−3/−4^ redox probe [94]. According to CV analysis, the thickness of the poly-pyrrole on the surface of the electrode was found to increase with increasing scan cycles, and the peak current also increased. The quantification of the thickness of the poly-pyrrole on the surface of the electrode was measured using Faraday’s law using the charge that was due to pyrrole electrooxidation during cyclic voltammetry. This could be attributed to poly-pyrrole being a conductive polymer. EIS showed a change in the electron transfer resistance, which is dependent on the dielectric and insulating features of the electrode/electrolyte interface. The change in mass transfer was controlled by the electron transfer kinetics of [Fe(CN)_6_]^−3/−4^, as the authors noted a huge mass transfer control with increasing scan rates. When the polymer film layer was increased, the rate of charge transfer was found to decrease [94].

**Figure 2:**
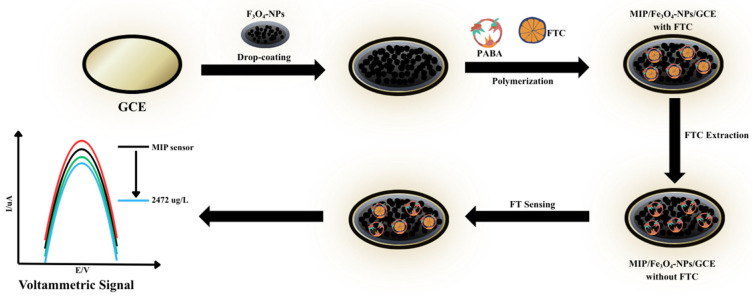
Schematic representation of MIP/Fe_3_O_4_ NPs/GCE sensor fabrication (Adapted from [95]).

Mokwebo and colleagues [95] fabricated and characterized an electromimetic molecularly imprinted polymer sensor for the detection of emtricitabine in wastewater [96]. It was attained by electropolymerization of poly-(para-aminobenzoic acid) (functional monomer) and the emtricitabine (template) using cyclic voltammetry at GCE (Figure 2). Prior to electropolymerization, a layer of iron oxide nanoparticles was drop-coated onto the GCE, forming a thin film.

The emtricitabine was found to block the electron transfer of poly-(para-aminobenzoic acid) during the electropolymerization. Since emtricitabine is a non-electroactive species, a decrease in the peak current was observed before template extraction. The sensor’s response was enhanced (increased peak current) after the template extraction. DPV analysis was used to generate a calibration curve in a linear range of 1.24 to 24.7 μg/L, resulting in limits of detection and quantification of 0.439 and 1.30 μg/L, respectively. The sensor was applied to real wastewater samples (influent and effluent); the samples were spiked with 12.36 and 247.25 μg/L. A recovery range of the emtricitabine (analyte) was from 98.8 to 101.5% [95]. The fabricated electromimetic molecularly imprinted polymer sensor showed good sensitivity within a suitable linear range, and high recoveries, which indicate good accuracy and practical application since it was applied even on complex matrix samples (wastewater influent and effluent) [95].

**Figure 3:**
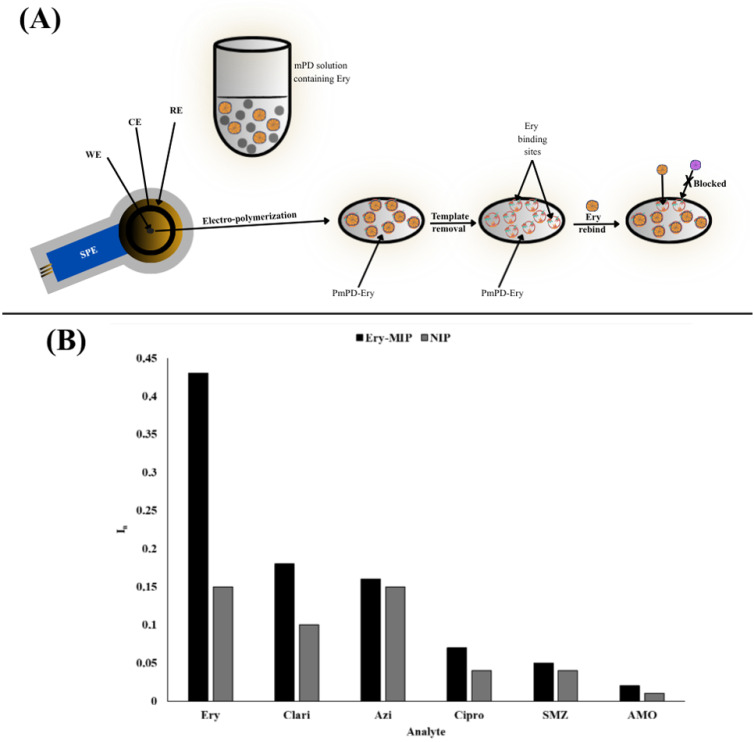
(**A**) The schematic diagram of the fabrication of the Ery-MIP film on the gold working electrode of SPE; (**B**) DPV responses resulting from incubating Ery-MIP and NIP modified SPEs in 40 μM analyte concentration in PBS of each different antibiotic (SMZ, AMO, Cipro, Azi, Clari, and Ery) (Adapted from [96]).

Ayankojo et al. [96], fabricated a portable electrochemical sensor for effective detection of erythromycin (Ery) macrolide, known as an emerging water contaminant. The sensor was achieved (Figure 3A) by combining the MIP synthesis with the screen-printed electrode via electropolymerization. A constant potential of 0.63 V was applied to the gold surface of the working electrode of the screen-printed electrode, which was submerged in an aqueous solution containing a functional monomer, template molecule, and phosphate buffered saline as an electrolyte. After the polymer film deposition, the template molecule was then removed, leaving binding sites specific to erythromycin (Ery) rebinding. The resulting electrochemical sensor was characterized using electrochemical impedance spectroscopy (EIS) and cyclic voltammetry (CV). The EIS studies were conducted at an alternating amplitude of 10 mV and a 0.1 to 100 kHz frequency range in the presence of 1 M potassium chloride (KCl) and 4 mM redox probe K_3_[Fe(CN)_6_]/K_4_[Fe(CN)_6_]. The CV studies were performed in the same solution as the redox probe, but a potential range between 0 and 0.5 V was applied at a scan rate of 50 mV/s. Rebinding and selectivity experiments were carried out using the differential pulse voltammetry (DPV) technique, which was performed in the same solution as the redox probe at a potential range of 0 to 0.4 V with pulse amplitude and width of 0.025 V and 0.1 s, respectively. Different functional monomers were investigated, as was their binding energy (interaction between monomer and erythromycin). The following binding energies of the monomers were 60.2, 119.2, 130.5, 202.7, 396.6, 407.9, and 416.0 kcal/mol for pyrrole (PY), 2-mercaptonicotinic acid (MNA), 1H-pyrazolo [3,4-b]pyridine-3-carboxylic acid (Pyrazo), dopamine (DA), 1,8-diaminonaphthalene (1,8-DAN), m-phenylenediamine (mPD), and 2-aminopyridine (2-AP), respectively. Both 2-aminopyridine (2-AP) and m-Phenylenediamine (mPD) had the highest binding energy compared to other monomers. The m-Phenylenediamine (mPD) was chosen as a suitable monomer since 2-AP polymer films deposited onto the surface of the transducer were highly unstable when exposed to organic solvents compared to mPD. The sensors showed a high affinity for erythromycin (Ery) in selectivity studies (Figure 3B) that included other interfering antibiotics (azithromycin (Azi), clarithromycin (Clari), ciprofloxacin (Cipro), sulfamethizole (SMZ), and amoxicillin (AMO). The signals induced by the sensor when incubated in a solution containing each analyte were observed. The erythromycin (Ery) signal was 3 times higher than those of azithromycin (Azi) and clarithromycin (Clari), while the erythromycin (Ery) signal was also 9 times, 29 times, and 46 times higher than those of sulfamethizole (SMZ), ciprofloxacin (Cipro), and amoxicillin (AMO), respectively. The developed sensor showed a good analyte recovery range of 91–102%. The limit of detection (LOD) and limit of quantification (LOQ) observed were 0.1 nM and 0.4 nM, respectively [96].

Aswini and co-workers [97] developed an MIP-based electrochemical sensor for the detection of amino acid (L-cysteine (Cys)) by using methacrylic acid as a functional monomer in the formation of molecularly imprinted polymer (MIP)-modified carbon paste electrode (CPE). Ethylene glycol dimethacrylate (EGDMA) (cross-linker), azobisisobutyronitrile (AIBN) (initiator), and methanol as the porogen solvent were used during polymerization of the MIP. Then the MIP was combined with graphite powder, paraffin oil, and a copper wire to form a transducer MIP-CPE. Characterization was done using Fourier transform infrared, cyclic voltammetry, and differential pulse voltammetry, the optimum condition was described in terms of % composition of MIP in MIP-CPE as well as the pH. According to DPV response 25% of the MIP composition in MIP-CPE and a pH of 7 were found to be the optimum conditions. Cyclic voltammetry, differential pulse voltammetry, and impedance analysis of the MIP-CPE response showed the detection of amino acid with an LOD of 9.6 nM and a linear correlation coefficient of 0.9974 (linearity), and the detected concentration range of L-cysteine was 2 × 10^−8^ to 18 × 10^−8^ M from tap water [97].

Arshad et al. [98], developed a molecularly imprinted polymer (MIP) based on an impedimetric sensor for the detection of dengue (a viral infection). The MIP was prepared using dopamine as a functional monomer, which was polymerized (coated) directly on the polysulfone nanofiber-modified screen-printed carbon electrodes (SPCE) in the presence of a template (dengue non-structural protein 1 (NS1)). The porogen solvents used were tetrahydrofuran and dimethylformamide in a ratio of 3:1. The template was removed by washing the electrode with PBS, and other impurities were also washed with sulfuric acid. The electrochemical properties of the NS1 MIP-based impedimetric sensor were investigated by cyclic voltammetry and electrochemical impedance spectroscopy. The electrochemical measurements of the sensor were conducted at pH 7.4 of the PBS, which contains a ferricyanide/ferrocyanide couple as the redox probe. A better redox peak was obtained at 50 mV/s from the voltammograms when a different potential range (−1 to +1 mV) was applied to the sensor. Cyclic voltammetry shows a slow electron diffusion process for the flow of electrons, which increased as the potential energy was increased. This was initiated by the insulation caused by the presence of NS1. A linear response within the concentration range of 1 to 200 ng/mL was determined by the impedimetric measurements, while the limit of detection was 0.3 ng/mL at optimum conditions. Spiked samples which contain all the interfering species (including albumin, globulins, and fibrinogen) were investigated via impedimetric signals of MIP and NIP. ΔRatio values calculated from impedimetric signals response showed higher values for NS1 compared to the NIP and other interferences [98].

Even though there is an increase in the development of electrochemical sensors based on molecularly imprinted polymers for pharmaceutical drugs in environmental and biomedical fields [99], there is very limited work reported on their application in wastewater samples. This concern is significant because wastewater treatment plants are the main contributors to the accumulation of pharmaceutical drugs in the environment [100].

## 7. Application of MIPs Based on Electrochemical Sensors for the Detection of Pharmaceutical Drugs in Various Sample Matrices

Table 3 provides an overview of various molecularly imprinted polymers (MIPs) used with electrochemical methods for the detection of pharmaceutical drugs in various sample matrices. Electrochemical techniques coupled with MIPs, known for their exhibiting operational simplicity, inexpensive preparation, rapid response, low detection limits, high sensitivity, selectivity, and versatility, have become important tools in pharmaceutical analysis. The table looks at the approaches, from voltammetry to electrochemical impedance spectroscopy (EIS), each suited for specific drug detection challenges in matrices such as water, cosmetic products, serum, urine, food produce, and many others. By taking into account these techniques in future wastewater analysis, we will be able to better employ their applicability, advantages, and limitations in wastewater testing. This comparison highlights the growing significance of molecularly imprinted polymer-based electrochemical sensors in ensuring accurate and reliable drug monitoring in wastewater treatment plants.

Table 3 suggests that MIPs combined with electrochemical sensors can be successfully applied to different and complex matrices and can be able to produce high selectivity and sensitivity since very low detection limits were observed for the targeted analytes. Therefore, MIP-based electrochemical sensors hold huge potential for the detection of pharmaceuticals in wastewater, since wastewater consists of a complex matrix with many interfering substances.

Water pollution by pharmaceutical compounds is of huge concern since the water quality decreases drastically, posing risks through the consumption of the polluted water. Constant consumption of contaminated water can lead to health problems including exposure to drug-resistant diseases, and endocrine disruption in humans and animals, and can harm the aquatic ecosystem [109]. Therefore, it is important to develop appropriate sensors for detection and monitoring of pharmaceutical compounds in our water systems. This will help eliminate the problem by identifying the sources of pollution, assessing the potential risks, and instituting implementation of the mitigation strategies to improve the water quality.

## 8. Concluding Remarks

This review has provided a comprehensive assessment of design strategies, and applications of molecularly imprinted polymer (MIP)-based electrochemical sensors, with particular emphasis on their use in detecting pharmaceutical contaminants in wastewater. By integrating advances in imprinting techniques, sample matrices, and electrochemical transduction methods, these sensors have demonstrated significant potential as selective, sensitive, and cost-effective analytical platforms for environmental monitoring.

Despite these advances, several critical challenges continue to limit their widespread application and commercialization. Issues such as template leakage, non-specific binding, electrode surface fouling, and matrix interferences remain major obstacles, particularly in complex real-world samples. In addition, limited long-term stability and difficulties associated with template removal without compromising binding site integrity further constrain sensor reusability and reliability.

From a materials perspective, while noble metal and advanced carbon-based electrodes offer excellent performance, their high cost restricts large-scale deployment. In this context, carbon paste electrodes emerge as a promising alternative due to their low cost, ease of fabrication, and tunable composition, enabling improved control over MIP loading and binding site density. However, further research is required to fully exploit their potential, particularly in combination with nanomaterials.

The incorporation of nanostructured materials has significantly enhanced sensor performance by improving conductivity, surface area, and electron transfer kinetics. Nevertheless, systematic studies on the integration of nanomaterials with MIP-based carbon paste electrodes remain limited, representing an important direction for future research. Furthermore, the development of portable, field-deployable sensing devices based on these platforms is essential for real-time, on-site monitoring of pharmaceutical pollutants.

Looking ahead, future efforts should focus on improving sensor reproducibility, minimizing matrix effects, and enabling multi-analyte detection in complex environmental samples. Advances in green synthesis, low-cost materials, and scalable fabrication strategies will be crucial for translating laboratory-scale developments into practical technologies. Ultimately, the continued development of robust and affordable MIP-based electrochemical sensors holds significant potential for supporting environmental surveillance and informing regulatory strategies aimed at mitigating pharmaceutical pollution in water systems. Such advancements will be instrumental in bridging the gap between laboratory-scale innovation and real-world implementation of sustainable water quality monitoring systems.

## Figures and Tables

**Table 1:** Overview of molecularly imprinted polymers (MIPs): sensing technologies, target analytes, chemical mechanisms, advantages, and limitations.

Sensing Technology	Target Analytes	Chemistry/Mechanism	Advantages	Drawbacks	References
Differential pulse voltammetry (DPV) within MIP voltammetry	Pharmaceutical and trace pollutant detection.	Pulse technique measures current differences before and after pulse application.	Most sensitive voltammetry technique for trace analysis.	Sensitive to electrode fouling and matrix effects.	[5]
MIP-based potentiometric sensors	Electrolyte ions, heavy metals, pharmaceuticals, and biomolecules.	MIPs are added to function as synthetic ionophores in ion-selective electrodes (ISEs), converting ion activity into electrical potential according to the Nernst equation.	Enhanced selectivity and sensitivity for organic/biological ions, simple instrumentation operation, and cost-effectiveness.	Organic analytes tend to require protonation/deprotonation buffers and are limited to certain types of synthetic receptors/MIPs.	[60]
MIP-based voltammetric sensors, (CV, DPV, SWV, ASV, and LSV)	Drug analysis, environmental monitoring, etc.	MIP-based voltammetric measures faradaic current produced during redox reactions.	Improved sensitivity, direct electrochemical sensing, etc.	Electrode passivation and fouling is the issue, and non-electroactive analytes require redox probes.	[5,61]
MIP-based impedimetric sensors	Monitoring template removal, analyte rebinding, etc.	Electrochemical impedance spectroscopy (EIS) measures capacitance and charge-transfer resistance changes at the MIP-modified interface.	Label-free (any response caused by analyte binding) detection, very sensitive to slight changes happening at the surface of the electrode, and useful for MIP characterization.	Interpretation may be complex. Sensitive to fouling and non-specific adsorption.	[61,62]
MIP-based optical sensors	Surface plasmon resonance, fluorescence, interferometric sensing, etc.	Detect optical changes occurring when analytes bind to MIP recognition sites.	Broad detection window; high specificity; suitable for biomolecules.	Require expensive instruments, and some methods need labels/markers (e.g., fluorescent dye, an enzyme, a radioactive tag) to work.	[63]
MIP-based drug delivery systems (DDS)	Controlled therapeutic delivery, targeted cancer therapy, etc.	MIPs encapsule drugs within polymer matrices and regulate controlled release through selective interactions.	Sustained drug release; targeted delivery; reduced side effects; high drug-loading capacity.	Long-term biocompatibility and toxicity still remain an issue due to insufficient research.	[64,65]
MIP-based catalysis	Biomimetic catalysis and selective synthesis.	MIPs mimic enzyme active sites by imprinting transition states or intermediates into polymer cavities.	High stability; selective catalysis; enzyme-like specificity.	Lower catalytic efficiency compared to natural enzymes.	[66,67]
MIP-based separation techniques	Wastewater purification, food analysis, biological sample preparation.	MIPs act as selective sorbents for extraction and purification processes.	High selectivity; reusable; stable in harsh acidic/basic conditions and complex matrices.	Template leakage and incomplete analyte recovery may occur.	[68]

**Table 2:** Different types of matrix effects from commonly present components in environmental samples which impact the performance of the sensor.

Matrix Components	Effect on Sensor Performance	References
Competing pharmaceuticals such as structural analogs, co-contaminants	Competition can impact selectivity of the MIP since competing analogs can occupy recognition sites and impact the calibration slopes (method validation). The probe diffusion/transport can be altered. The sensor’s response becomes hindered due to binding of non-target species, resulting in reduced analytical specificity.	[71]
Salts/high-ionic strength ions such as Na^+^, Ca^2+^, Cl^−^, SO_4_^2−^	These components can mask the attractive forces (hydrogen bonding and electrostatic interactions) between the template and MIPs binding sites. The effect of salt can either increase or decrease diffusion of redox probe or electric double layer thickness (charged electrode surface) depending on the charge present. The sensor performance may improve sensitivity due to decrease in charge transfer due to compression of the charged electrode surface which enhances electron transfer. The sensitivity increases due to specific ion adsorption.	[72,73,74,75]
Transformation products such as metabolites, degradation products	The similarity of the chemical structure of analytes and metabolites can cause cross-reactivity, and binding sites can be occupied by either one. The electrode surface experiences fouling increase as metabolites accumulate, and changes in the charge transfer resistance can cause misinterpretation of the data.	[71,76]
Suspended solids such as clays, bioflocs, particulates	Suspended solids can impact MIP binding sites by physically blocking them. The analyte can mix with suspended solids, making binding hard since there is a reduced free concentration of the analyte. Suspended solids can also stop the diffusion of the redox probe (ferri/ferrocyanide) and enhance mass-transfer resistance. The charge transfer resistance can increase due to unstable electrode surface or fouling.	[76,77,78]
Dissolved organic matter such as humic/fulvic acids, proteins	Can cause recognition site blocking or partially occupy MIPs, impacting MIPs’ affinity for target analytes (selectivity). Can form an adsorbed organic layer on the transducer surface, causing an electrostatic interaction which blocks the diffusion of the redox probe. Sensor response can be altered by an increase in charge transfer resistance due to surface fouling, causing heterogeneity (inaccessibility) of binding sites.	[71,76,77,79]

**Table 3:** Application of MIPs based on electrochemical sensors for the detection of pharmaceuticals in different matrices.

Analyte	Monomer/Crosslinker/ Extraction Solution	Sensing Material	Sensor’s Method Type	Sample Matrix	Linear Range	Limits of Detection	References
Midazolam	MAA, EGDMA, methanol:acetic acid (8:2, *v*/*v*)	Carbon paste electrode (CPE)	CV, SWV, and DPV	Human urine	5.0 × 10^−10^ to 1.0 × 10^−7^ M and 1.0 × 10^−7^ to 1.0 × 10^−6^ M	1.77 × 10−10 M	[21]
2,4-dichlorophenol	Methacrylic acid (MAA), ethylene glycol dimethacrylate (EGDMA), methanol: water (1:1, *v*/*v*)	Graphene oxide (GO)-modified glassy carbon electrode (GCE)	CV and DPV	Water	0.004–10.0 μM	0.5 nM	[101]
Paraben	2 Hydroxyethyl methacrylate, ethylene glycol dimethacrylate (EDGMA), deionized water	Screen-printed gold electrodes (Au-SPE)	CV and SWV	Cream sample (cosmetic products)	1–30 μM	0.706 μM	[102]
Bisphenol-A	Pyrrole (Py), lithium perchlorate, acetonitrile	Graphene oxide-coated glassy carbon electrode (GCE)	CV	Water and milk	750–0.5 nM	0.2 nM	[103]
Amoxicillin	(3-Aminopropyl) triethoxysilane (APTES), phenyltriethoxysilane (PTES), anhydrous ethanol	Graphene oxide (GO)-modified glassy carbon electrode (GCE)	CV and DPV	Amoxicillin tablet dissolved in ethanol/water	5.0 × 10^−10^ to 9.1 × 10^−7^ M	2.94 × 10^−10^ M	[104]
Deltamethrin	Sodium acetate trihydrate ≥99.5%, o-phenylenediamine, methanol, ultra-pure water	Cobalt(III) oxide-coated indium tin oxide electrodes	CV and EIS	Straining tomato, salad, grapefruit, and orange samples	3.91 nM to 65.0 nM and	2.43 nM and 726.0 nM	[105]
Norfloxacin, Enoxacin, Pefloxacin, Enrofloxacin, Gatifloxacin	Methacrylic acid (MAA), ethylene glycol dimethacrylate (EGDMA), methanol:acetic acid (9:1, *v*/*v*)	3D-framework of functionalized multi-walled carbon nanotube (fMWCNT)-coated glassy carbon electrode (GCE)	CV, DPV, and EIS	Sprague Dawley rat serum	0.003–0.391 μM and 0.391–3.125 μM	1.58 nM	[106]
Isoproturon	Pyrrole, tetrabutylammonium tetrafluoroborate, Milli-Q water, ethanol/water (70:30 *v*/*v*), and sulfuric acid	GCE	CV and SWV	Water	0 to 5 × 10^−7^ M	1.1 × 10^−8^ M	[107]
Ciprofloxacin	Polyaniline − Poly(o-phenylenediamine)	Reduced graphene oxide (rGO)-modified glassy carbon electrode (rGO/GCE)	DPV	Surface water	1.0 × 10^−9^ to 5.0 × 10^−7^ M	5.28 × 10^−11^ M	[108]

## Data Availability

No new data were created or analyzed in this study.
